# The evolution and diversity of the nonsense-mediated mRNA decay pathway

**DOI:** 10.12688/f1000research.15872.2

**Published:** 2018-11-22

**Authors:** James P. B. Lloyd

**Affiliations:** 1 ARC Centre of Excellence in Plant Energy Biology, University of Western Australia, Perth, Australia

**Keywords:** RNA, NMD, evolution, UPF1, SMG1, transposable element, RNA decay

## Abstract

Nonsense-mediated mRNA decay is a eukaryotic pathway that degrades transcripts with premature termination codons (PTCs). In most eukaryotes, thousands of transcripts are degraded by NMD, including many important regulators of developmental and stress response pathways. Transcripts can be targeted to NMD by the presence of an upstream ORF or by introduction of a PTC through alternative splicing. Many factors involved in the recognition of PTCs and the destruction of NMD targets have been characterized. While some are highly conserved, others have been repeatedly lost in eukaryotic lineages. Here, I detail the factors involved in NMD, our current understanding of their interactions and how they have evolved. I outline a classification system to describe NMD pathways based on the presence/absence of key NMD factors. These types of NMD pathways exist in multiple different lineages, indicating the plasticity of the NMD pathway through recurrent losses of NMD factors during eukaryotic evolution. By classifying the NMD pathways in this way, gaps in our understanding are revealed, even within well studied organisms. Finally, I discuss the likely driving force behind the origins of the NMD pathway before the appearance of the last eukaryotic common ancestor: transposable element expansion and the consequential origin of introns.

## What is nonsense-mediated mRNA decay?

Gene expression is controlled by a variety of mechanisms, sometimes in unexpected ways. Analysis of mutant screens and genetic diseases identified mutations that introduced nonsense mutations, but surprisingly, these premature termination codons (PTCs) lead to a reduction in mRNA stability ^1, 2^. This increase in RNA decay is the result of an active translation-dependent process ^1, 3^. This pathway was termed nonsense-mediated mRNA decay (NMD) and is now known to regulate hundreds to thousands of transcripts in plants, animals, fungi and ciliates ^4– 10^. Many of the NMD targeted transcripts are not the result of nonsense mutations, but are instead the result of alternative splicing events that introduce PTCs or the presence of an upstream open reading frame (uORF). Many such splicing events are not the result of splicing errors, but are in fact highly conserved events ^11, 12^. Therefore, NMD has a major role in shaping the transcriptome of diverse eukaryotes. However, the exact molecular nature of the NMD pathway varies between organisms. Most eukaryotes share the core NMD factors (see below), but an impressive number of modifications to the NMD pathway exist. In this review, I will examine the factors known to act in NMD, discuss the diversity of these factors in eukaryotes, and explore the different mechanisms that explain how a PTC is differentiated from an authentic stop codon. Finally, I will discuss how the NMD pathway may have evolved and some remaining key questions in our understanding of the NMD pathway.

## The factors that read nonsense

**Figure 1.**
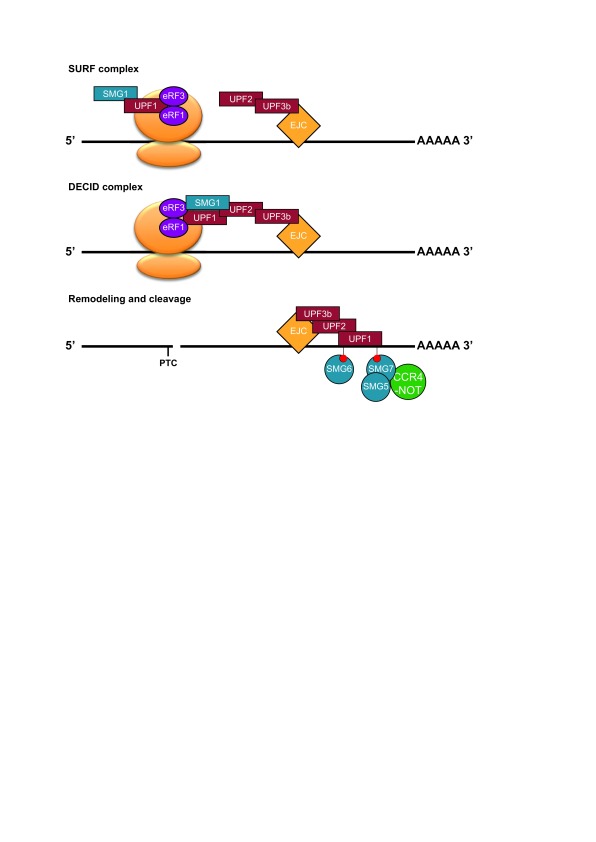
The model of NMD activation in animals. At termination events, UPF1 and SMG1 are recruited to termination events by eRF1 and eRF3, leading to the formation of the SMG1-Upf1-eRF1-eRF3 (SURF) complex ^22^. Further recruitment of UPF2 and UPF3 (UPF3b in mammals) leads for the formation of a decay-inducing (DECID) complex ^20, 46^. This will lead to the phosphorylation of UPF1 by SMG1. Then the ribosome will disassociate and SMG5/6/7 will be recruited to transcript through phos-UPF1 binding. The transcript is degraded by endonucleolytic cleavage by SMG6 and the CCR4-NOT complex is recruited by SMG7/5. UPF2 and UPF3 can be recruited to NMD targeted transcripts by the EJC, although many transcripts a degraded without the presence of an EJC ^47, 48^.

Early mutant screens in baker's yeast and *Caenorhabditis elegans* identified three conserved factors that could suppress a nonsense mutation ^13, 14^. These factors were named UP-frameshift (UPF) 1, 2 and 3 in baker's yeast and Suppressors with Morphological defects on Genitalia (SMG) 2, 3 and 4 in *C. elegans*. The baker's yeast names of these factors are used throughout this review. UPF1 is a highly conserved RNA helicase ^15^ that interacts with UPF2, which is an MIF4G domain-containing protein ^16^, that in turn binds to UPF3 ( Figure 1) ^17, 18^. The initial mutant screens in *C. elegans* also revealed four additional factors: the kinase SMG1 and the 14-3-3-like domain proteins SMG5, SMG6 and SMG7 ^13, 19^. In animals, SMG1 is known to phosphorylate UPF1 after a PTC is been recognised ( Figure 1) ^20– 22^. From these early studies in *C. elegans*, the different NMD factors were defined by their role in the phosphorylation of UPF1. UPF2 and UPF3 support the phosphorylation of UPF1 by creating a complex compatible for phosphorylation by SMG1 ^22^, while also acting to activate the RNA helicase activity of UPF1 ^23^. SMG5/6/7 bind to phosphorylated UPF1 ^24^ and are active in the dephosphorylation of UPF1 by recruiting the PP2A phosphatase ^25– 27^. However, it is now clear that their primary role is in acting at various stages of RNA decay. SMG5/6/7 have a central role in recruiting the degradation machinery to degrade the NMD target ^28– 31^ ( Figure 1). SMG5 and SMG7 act to recruit exonucleases ^29^, while SMG6 is an endonuclease, cutting the transcript near the PTC ^30, 31^. Over time, many more NMD factors have been identified through further genetic and biochemical screens ^32– 35^. Of these, SMG8 and SMG9 are of particular interest. First identified in human cells as SMG1-interacting proteins, they act in the NMD pathway of humans and possibly *C. elegans* ^34, 36^ through the inhibition of the kinase SMG1. Curiously, studies in mammals have revealed that many NMD targets do not require the involvement of all NMD factors. Many NMD targets are degraded by specific “branches” of the NMD pathway that do not require UPF2 ^37^ or UPF3b ^38^ in mammals. However, all branches do involve UPF1, highlighting its central importance to the NMD pathway.

Together these studies, mostly using animal systems, paint a picture where multiple factors (UPF2, UPF3, SMG1, SMG8, and SMG9) assist in the activation of UPF1, while other factors (SMG5/6/7) act to degrade an NMD target and dephosphorylate UPF1.

## Variations on a common pathway

**Figure 2.**
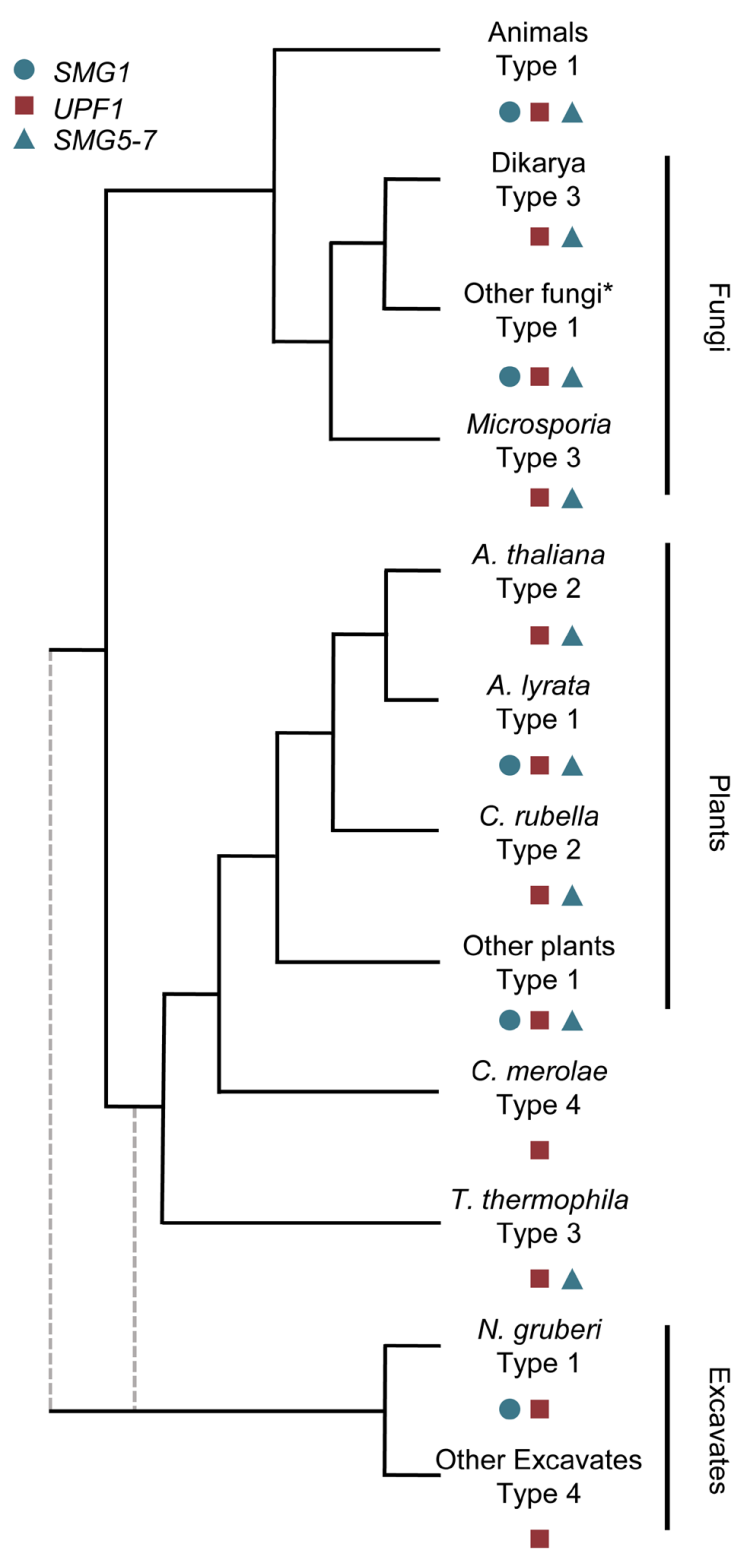
The various NMD types across diverse eukaryotic lineages. The distribution of the key NMD factors, UPF1, SMG1 and a member of the SMG5-7 family define the NMD pathway type. All NMD types have arisen multiple times within eukaryotic evolution. NMD pathways can be classified into four types, Type 1: classical SMG1 dependent NMD, Type 2: recent loss of SMG1 with S/TQ rich UPF1, Type 3: ancient loss of SMG1 with S/TQ depleted UPF1, Type 4: Heavily derived NMD ( Figure 3). To date, no SMG5/6/7 family member has yet been identified in *N. gruberi* but given the presence of SMG1 ^45^, I am currently classifying it as a type 1 NMD pathway. The branch lengths do not reflect the relatedness of any species, but represent the order of separation between the lineages. The root of eukaryotes is unclear, so branches representing a Excavata early and late divergence are represent in grey, dashed-lines. *SMG1 appears to have been lost in other fungal lineages as well, representing repeated losses in multiple fungal lineages ^45^.

**Figure 3.**
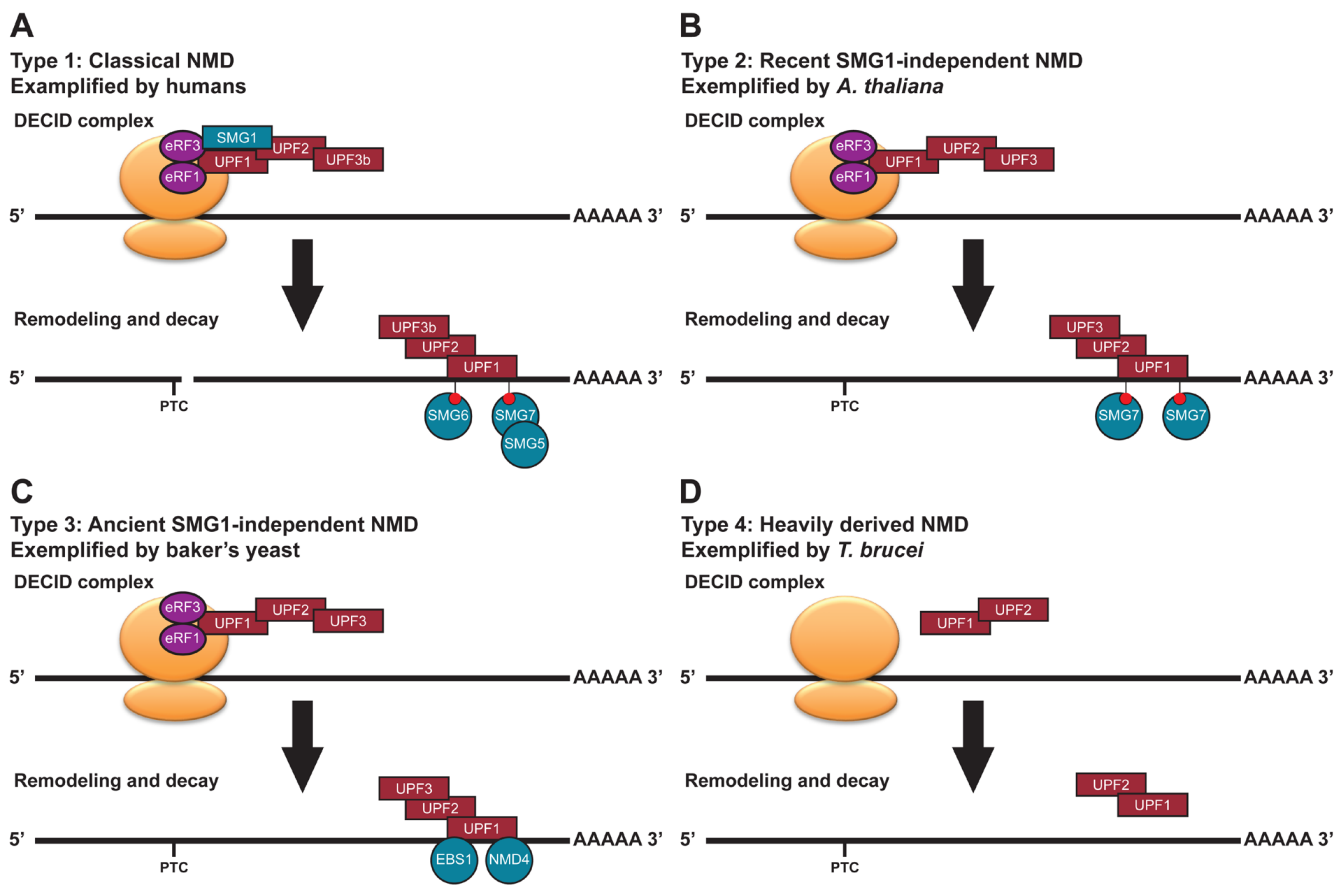
Models of evolutionarily diverse NMD pathways. ( **A**) Classical NMD, exemplified by humans (modified from Figure 1). ( **B**) Recent SMG1-independent NMD, exemplified by *A. thaliana*. *A. thaliana* lost SMG1 within the last 5–10 million years ^45, 51^. *A. thaliana* requires SMG7 for a functional NMD pathway ^50^, retains a S/TQ rich UPF1 ^45^ and its UPF1 needs to be phosphorylated to function in NMD in tobacco leaves ^53, 68^. This suggests an alternative kinase may have replaced SMG1. ( **C**) Ancient SMG1-independent NMD, exemplified by baker’s yeast. The NMD pathway of baker’s yeast was the first to be characterised. UPF1, UPF2 and UPF3 have central roles in this pathway. Reverse genetics revealed a potential lesser role for EBS1, a SMG7 homologue, in NMD ^60^ but its UPF1 is depleted in S/TQ dipeptides ^45^. ( **D**) Heavily derived NMD, exemplified by *T. brucei*. It is unclear if a functional NMD pathway exists in these organisms. In *T. brucei*, it has been shown that UPF1 and UPF2 interact, but their interaction with the ribosome and potential NMD targets is unclear ^43^. Tethering of UPF1 a transcript can decrease its abundance ^43^.

Despite the deduction of a basic schematic of the NMD pathway in animals ( Figure 1), many of the factors involved in this classical model of NMD vary between different organisms ( Figure 2 and Figure 3). The most highly divergent NMD pathways are those found in the excavata ( Figure 2 and Figure 3). The excavata have been suggested to be the most basal group of eukaryotes ^39^, although other work places them within the same supergroup as plants ^40, 41^. Although the NMD pathways of the parasites *Giardia lamblia* and *Trypanosoma brucei* have been studied, it is unclear if a functional NMD pathway exists in these organisms ^42, 43^. They contain heavily reduced compliments of NMD factors: the genome of *G. lamblia* only harbors UPF1, and the genome of *T. brucei* only harbors UPF1 and UPF2 ^42, 43^. Over-expression of UPF1 in *G. lamblia* caused an NMD reporter to further decrease, suggesting that *G. lamblia* might have an active NMD pathway ^42^. In contrast, the knockdown of UPF1 in *T. brucei* did not increase NMD reporter construct expression, or endogenous genes ^43^. However, tethering of UPF1 in *T. brucei* did decrease reporter expression ^43^. Therefore, it is difficult to definitively conclude the status of the NMD pathway in excavata. However, it is worth noting that parasites are known to have reduced genomes relative to free-living relatives ^44^, and that the non-parasitic excavata *Naegleria gruberi* does harbor the additional NMD factors of SMG1 and SMG9 ^45^. This indicates that a complex NMD pathway involving the kinase SMG1 likely existed in the last eukaryotic common ancestor.

Further support for a complex NMD pathway existing in the last eukaryotic common ancestor comes from the examination of plants. Plants, which diverged from animals and fungi early in eukaryotic evolution ( Figure 2), do have functional homologues of the NMD holy trinity: UPF1-3 ^47, 49^. Plants also have homologues of SMG5/6/7, known as SMG7 and SMG7-like ^50^, and SMG1 homologues ^45, 51^. SMG1 has been repeatedly lost throughout eukaryotic evolution, including two losses in land plants ( *Arabidopsis thaliana* and *Capsella rubella*) and multiple losses in fungi ( Figure 2) ^45, 51^. The repeated loss of SMG1 raises some interesting questions about the mechanism of NMD activation. In animals, and presumably in most plants, SMG1 phosphorylates SQ and TQ dipeptides at the N- and C-termini of UPF1 ^20, 52, 53^. Species, such as baker’s yeast, with an ancient loss of SMG1 ( Figure 2), have UPF1 sequences depleted of S/TQ dipeptides relative to species with SMG1 ^45^. Species that lost SMG1 more recently, such as *A. thaliana*, have UPF1 proteins that are rich in S/TQ dipeptides ^45^. The repeated losses of SMG1 in eukaryotes suggests that there is a genetic buffer, another factor/mechanisms that allows SMG1 to be lost but the NMD pathway to be activated ^45, 51^. In support of this notion, the experimental perturbation of SMG1 in fruit flies and zebrafish has little or no effect on the NMD pathway of these organisms ^54– 56^, suggesting that a backup UPF1-activation mechanism is already present in these species. One possibility is that an alteriave kinase has replaced SMG1 and might even be ancestral and operational in many species, allowing for the loss of SMG1 ^51^. However, this does not explain why the putative phosphorylation sites are lost in many species ^45^. One exciting possibility is that direct interactions between SMG5/6/7 family proteins is sufficient for NMD to be activated in some species (see below).

The SMG5/6/7 family split and diversified in the animal lineage, with the acquisition of the PIN domain in SMG5 and SMG6 ^27, 57, 58^. The PIN domain of SMG6 gives it the ability to act as an endonuclease, cutting the NMD targeted transcript near the PTC ^30, 31^. The SMG5/6/7 family also have a role in regulating telomere length ^59^. SMG5/6/7 homologues in plants are known as SMG7, given they lack the PIN domain of SMG5 and SMG6 ^50^. SMG5/6/7 family members of baker’s yeast, EBS1 and EST1, also lack the PIN domain ^60^. In baker’s yeast, EST1 is implicated in telomere regulation but not NMD, while a knockout of EBS1 reveals a mild NMD phenotype ^60, 61^. Given that baker’s yeast lacks SMG1 ^21, 45, 51^, it is not clear why EBS1/SMG7 would be required for NMD. The UPF1 of baker’s yeast is depleted of S/TQ dipeptides ^45^, which once phosphorylated by SMG1, normally act as binding site for SMG5/6/7 ^24^. The lack of S/TQ dipeptides suggest that classical phosphorylation of UPF1 is not required for the activation of NMD in baker’s yeast. Tyrosine phosphorylation of UPF1 in baker’s yeast has been observed and appears to regulate the RNA helicase activity of UPF1 ^62^, although the role in NMD, if any, and kinase responsible is still unknown. It is possible that these or other phosphorylated sites could act to recruit decay factors like S/TQ dipeptides do. However, given the differences between S and T residues from Y, it seems unlikely that EBS1/SMG7 would be involved. It could be that RNA decay enzymes are recruited directly to UPF1, alternative mechanism to the phosphorylation-mediated recruitment ^61, 63, 64^. Recently, the yeast EBS1 and NMD4 proteins were found to interact directly with UPF1 during NMD ^61^. NMD4, like SMG6, contains a PIN domain ^61^. Transcripts responsive to the deletion of UPF1 also increased in deletions of EBS1 and NMD4, however, to a lesser extent ^61^. Interestingly, the importance of EBS1 and NMD4 became more pronounced when yeast cells expressed a truncated UPF1 ^61^; when the truncated UPF1 was expressed alone, NMD efficiency was about 30% of wild-type, in contrast, when either EBS1 and NMD4 were deleted in the truncated UPF1 lines, NMD efficiency was close to zero ^61^. This suggests that EBS1 and NMD4 become essential in NMD limiting conditions. This raises the possibility that in species lacking SMG1, the phosphorylation checkpoint of NMD is not required and SMG5/6/7 family proteins directly interact with UPF1 when at a PTC. SMG1 mutants in fruit flies have been found to have a lesser effect on NMD than the mutation of other NMD factors ^54, 55^. SMG5 was found to be essential for NMD ^28^, and when a mild disruption of SMG5 is introduced, mutations of SMG1 enhanced the severity of the NMD phenotype ^28^. This supports the notion that NMD can be activated without phosphorylation and that phosphorylation simply enhances decay under limiting conditions ^28, 65^. Interestingly, mammalian SMG6 has also been found to bind UPF1 independent of phosphorylation ^66, 67^, suggesting some level of conservation of phosphorylation-independent recruitment of decay factors in NMD. However, it is not clear why a phosphorylation checkpoint is needed for NMD in some organisms like mammals ^20, 52^ and plants ^51, 53^, but likely not others such as yeast, but direct interaction seems likely to be the mechanism. Recently, another member of the SMG5/6/7 family was characterized in the ciliate *Tetrahymena thermophila* ^9^, despite the loss of the SMG1 kinase from *T. thermophila* ^9, 45^. The SMG56/7 family member of *T. thermophila* was named SMG6-like (SMG6L) due to the presence of the C-terminal NYN nuclease domain, potentially taking on the same role as the PIN domain of animal SMG6 proteins ^9^. SMG6L appears to work with UPF1 in the NMD pathway of *T. thermophila* and is conserved in many other protozoa ^9^. However, it is unclear if SMG6L directly interacts with UPF1 or if it is via phosporylation, but there is no SMG1 and classical phosphorylation sites on UPF1 ^9^.

The kinase activity of SMG1 is regulated in part by SMG8 and SMG9 ^34^. These factors have been identified but not characterized outside of the animal kingdom ^45^; a curious finding which indicates they may have a role in NMD in diverse eukaryotes. When SMG1 is lost from a genome, SMG8 and SMG9 are generally also lost ^45^. Further work will be needed to reveal the extent of any conserved role in NMD for these factors.

Taken together, a diverse set of NMD pathways with varying levels of classically defined NMD factors been identified. Generally speaking, these can be split into four major types and a spread across many unrelated eukaryotic lineages ( Figure 2 and Figure 3):

**1)** Classical SMG1-dependent NMD (As exemplified by humans, worms, and moss)
**2)** Recent SMG1-independent NMD (As exemplified by *A. thaliana*)
**3)** Ancient SMG1-independent NMD (As exemplified by baker’s yeast and *T*. *thermophila*)
**4)** Heavily derived NMD (As exemplified by *G*. *lamblia*, *T*. *brucei* and *Cyanidioschyzon merolae)*

Type 1 NMD pathways (classical SMG1-dependent NMD; Figure 3A) are known to exist in both animals and plants ^20, 21, 51^ and is likely to be the ancestral state of NMD ^45, 51^. However, even here, the dependence on SMG1 is not always clear: SMG1 mutants in fruit flies have much milder phenotypes than mutations in other NMD factors ^55, 69^ and knockdown of SMG1 in zebrafish revealed no phenotype ^56^. It is possible that the NMD pathways of some species with a type 1 NMD pathway in appearance might better resemble type 2 NMD (recent SMG1-independent NMD).

Type 2 NMD pathways (recent SMG1-independent NMD; Figure 3B), such as those of the land plants *A. thaliana* and *C. rubella*, appear very much like those of type 1, with the exception of SMG1 being absent from the genome, likely with the accompanying loss of SMG8 and SMG9 ^45, 51^. However, UPF1 still maintains the relatively high level of phosphorylatable S/TQ motifs ^45^, and phospho-UPF1 binding protein SMG7 ^8, 50^. It would be tempting to speculate that a kinase related to SMG1 replaced it in the NMD pathway ^51^. ATM and ATR are two kinases from the same family as SMG1 that are conserved in plants and are involved in DNA repair. However, in *A. thaliana*, the reported mutant phenotypes of ATM and ATR ^70^ do not overlap with the classical NMD phenotypes ^49^, so this seems unlikely to be the case. TOR is the only other related kinase in *A. thaliana*, and is involved with the regulation of translation, although the phenotype of TOR knockdown lines do not appear to match those of NMD factors in *A. thaliana* ^71^.

A type 3 NMD pathway (ancient SMG1-independent NMD; Figure 3C), was the first to be characterized by a mutant screen in baker’s yeast ^14, 72^. These ancient losses of SMG1 lead to an NMD pathway without SMG1, without SMG8 and SMG9 ^45^, with a UPF1 depleted in S/TQ dipeptides ^45^, but a potential role for SMG5/6/7 proteins ^9, 60, 61^. Future work (see below) will be needed to better understand the exact molecular role of SMG5/6/7 proteins in type 3 NMD pathways, and to understand how the NMD pathway functions without the SMG1 activating UPF1.

Type 4 NMD pathways (heavily derived NMD; Figure 3D) are the most variable group and are found throughout the eukaryotic tree of life. These pathways often lack SMG1, but also core NMD factors (UPF2 and UPF3). Although UPF3 is hard to identify with homology searches ^73^, it does appear to be missing from the genomes of a number of species ^45^. These include the excavata parasites *G. lamblia* and *T. brucei* ^42, 43^ but also the red algae *C. merolae* ^51^. *C. merolae* has a very reduced genome, with only 27 introns in total ^74^. *C. merolae* and *G. lamblia* also lack homologues of UPF2. It is certainly possible that the presence of these factors do not represent a fully functional form of an NMD pathway and instead reflect the molecular reminance of a former NMD pathway whose factors have now been co-opted for other functions. NMD factors do function in other pathways, for example, UPF1 is known to be involved with mammalian DNA replication ^75^. Although in mammals, some NMD transcripts only require a subset of NMD factors ^37, 38, 76^, these branches of the NMD pathway support the notion that a more reduced NMD pathway may exist.

In any of these species, additional NMD factors are likely to have arisen. The only non-type 1 species to have had a forward genetics screen performed for is the baker’s yeast, so we have limited unbiased studies to draw from. Protein-protein interaction studies in yeast have revealed the species specific factor NMD4 ^61, 77^. Performing similar work in other species is likely to reveal more species/lineage specific factors. This will be especially exciting in type 4 species, with the most heavily reduced NMD pathways. This framework of NMD types based on presence/absence of conserved NMD factor is aimed at aiding the comparison and discussion of NMD pathways from diverse organisms. Thinking of all NMD pathways as being fundamentally the same at the molecular level is wrong. There is certainly an overlap, but more focused studies are needed to understand when homologous NMD factors do have the same molecular role in NMD and do not.

## Defining NMD targets

So far I have discussed the molecular processes that link the recognition of a PTC to transcript destruction. However, a lot of work has also been focused on understanding the mechanism of how a PTC is differentiated from an authentic stop codon. Multiple models for how this is achieved have been proposed. One of the most well characterized models centres around the exon junction complex (EJC), a protein complex deposited on an mRNA after two exons are ligated together during splicing ^78, 79^. While most EJCs are removed from the transcript by the translating ribosome ^80^, EJCs associated with exon-exon junctions ≥50 nt downstream of a stop codon are not removed and can elicit NMD ^81, 82^. Early work showed that the EJC was not involved in the NMD pathways of fruit flies ^58^, but more recent work proved the contrary, revealing a role for the EJC in fruit fly NMD ^83^. The EJC has been lost from baker’s yeast and so cannot have a role in its NMD pathway, but the EJC is involved in the fungi Neurospora crassa’s NMD pathway ^84^. The EJC mode has even found support in plants, with reporter genes and transcriptome-wide studies supporting a role for exon-exon junctions in 3’ UTRs eliciting NMD ^47, 85– 87^. These findings would suggest that the EJC mode is an ancient mechanism for targeting transcripts to NMD. A surprising version of the EJC mode is the finding that some NMD targets in *T. thermophila* appear to be dependent on splice junctions downstream of the stop codon, but not on the EJC itself ^9^. Knockout of the core EJC component Mago nashi did not alter the expression levels of NMD targets identified by knockout of UPF1 and SMG6L ^9^. This indicates that an alternative mechanism might maintain an EJC-like mode of NMD in *T. thermophila*.

Another well-explored system used in defining PTCs is the long 3’ UTR mode. Transcripts with abnormally long 3’ UTRs have been found in reporter genes ^47, 85, 88^ and transcriptome-wide studies ^5, 86, 89^ to target transcripts to NMD, although some recent transcriptome-wide studies found little to no trend across the transcriptome, when the presence of 3’ UTR introns were taken into account ^9, 87, 90, 91^. One proposed mechanism is the increased distance between the stop codon (PTC) and the polyA-binding protein, bound to the polyA tail ^92, 93^. This physical separation between the polyA tail and the terminating ribosome might lead to aberrant termination and the recruitment of NMD factors ^92, 93^. Although some transcripts appear to be targeted due to their length independent of the polyA tail in yeast ^94^. An alternative, but not mutually exclusive model posits that longer 3’ UTRs are able to recruit more UPF1 directly bound to the 3’ UTR ^95^. It has been found that UPF1 coats transcripts but translation displaces UPF1 from all regions, except the 3’ UTRs ^96^. This model suggests that a higher level of UPF1 binding increases the chances of NMD being triggered during the termination of translation; naturally long 3’ UTRs that are resistant to NMD have been observed to bind less UPF1 than susceptible long 3’ UTR transcripts ^95^. In fact some naturally long 3’ UTR transcripts in mammals appear to be protected from NMD by various features such as a recently identified cis-sequence element in the TRAM1 gene ^97^ or the many genes found to bind PTBP1 near the stop codon to prevent NMD ^46^. In yeast, the RNA binding protein Pub1 binds to sequence elements and protects some uORF-containing transcripts from NMD ^98^. Such features protecting long 3’ UTR transcripts from NMD might explain why transcriptome-wide studies find so few long 3’ UTR transcripts that are targeted to NMD.

The mechanisms used to define PTCs in the last eukaryotic common ancestor are unclear. While the EJC mode has been identified in plants, fungi, and animals, suggesting an ancient origin, there are many eukaryotic lineages where it has not been characterized, or does not function ^9, 99^. The long 3’ UTR mode of NMD has also been characterized in many diverse eukaryotes (plants, animals and fungi), but the mechanism underlying this mode, and failure to observe a strong signal for this feature in transcriptome-wide studies, does raise questions.

## The origins of NMD

Today, eukaryotes appear to utilize NMD in a variety of ways to achieve the same aim, degrading PTC-containing transcripts from a variety of sources. It appears that a rather complex NMD pathway, belonging to the type 1 group, existed in the last eukaryotic common ancestor (see above). In extant diploid eukaryotes, NMD can prevent some mutations from being dominant, protecting heterozygous individuals by turning these alleles recessive ^13, 100, 101^. However, NMD also increases the severity of some genetic disorders ^102^, creating a double-edged sword: protecting some mutation-carrying individuals while exacerbating the conditions of others. Therefore, it is unlikely that protecting the genome from nonsense mutations was the driving force behind the origin of the NMD pathway. Early eukaryotes did face a particular selective pressure not present in prokaryotes: rapidly multiplying transposable elements (TEs). The origin of sex in eukaryotes allowed for TEs to expand in copy number, which is not possible in prokaryotes with their primarily asexual reproductive system ^103, 104^. With the advent of sex, eukaryotes faced the expansion of many TE classes, including the self-splicing (group II) introns. Expansion of group II introns has been proposed to have driven the evolution of the spliceosome to enhance the splicing of these selfish elements ^105^, the nucleus evolved to physically separate the processes of transcription and translation and allow for intron removal before translation ^106^, and NMD evolved to degrade intron-retaining transcripts that escaped the nucleus ^106, 107^. These adaptations ensure that transcripts with retained introns do not undergo multiple rounds of translation. More recent expansions of introns in some eukaryotic lineages are due to the expansion of DNA transposons ^108^, indicating the importance of these mechanisms to protect the genome from TE expansions in extant eukaryotes, and suggests multiple origins for introns from TEs throughout eukaryotic evolution. NMD has been proposed as a general protection mechanism against RNA viruses and TE expansion ^109^. Once functional as a TE-intron protection pathway, NMD appears to have been co-opted to control gene expression. Today, in addition to repressing the expression of uORF-containing genes, pseudogenes and the products of alternative splicing, NMD may allow for the evolution of new introns. The presence of NMD may act as a buffer for novel introns with weak splice sites ^107, 110^. In fact, the red algae *C. merolae* only has 27 introns ^74^ and is missing all of the classical NMD factors with the exception of a UPF1 homologue ^51^. It is possible that *C. merolae* lacks a functional NMD pathway and this limits the acquisition of new introns, at least partly explaining its intron depleted genome.

## Unanswered questions

Many years of study have revealed diverse NMD pathways, centering on UPF1. However, there are a number of fundamental questions remaining in the field regarding the mechanisms and evolution of NMD.

**1)** Why is SMG1 repeatedly lost in different lineages? Is there a backup mechanism to activate UPF1 and is this conserved between the lineages that have recently lost (eg *A. thaliana*) and more anciently lost (eg baker’s yeast and *T*. *thermophila*) SMG1? Or are there multiple SMG1 replacement mechanisms?
**2)** What recruits the RNA degradation machinery to UPF1 when SMG1 is lost and S/TQ dipeptides are depleted? Does it rely on the direct interactions of SMG5/6/7 family proteins with UPF1 or another mechanism?
**3)** If phosphorylation of UPF1 represents a checkpoint in the activation of NMD, what explains the variability of the presence of this checkpoint between species?
**4)** Can the EJC mode of PTC recognition exist without the involvement of the EJC, potentially in *T. thermophila*? If so, what is the molecular basis for this and does it exist in other species?
**5)** What is the precise mechanistic roles of UPF2/UPF3 in relation to EJC mode and non-EJC mode NMD pathways? How do UPF2/UPF3 get recruited to NMD targets independently of the EJC?
**6)** To understand the discrepancies between transcriptome-wide and reporter construct approaches to the long 3’ UTR mode of NMD and to uncover the molecular mechanism(s) behind the long 3’ UTR mode.
7) To identify what precisely determines the accumulation of UPF1 on some transcripts, and why this appears to be dependent UPF1 ATPase activity ^111^.
8) What is the mechanism leading to NMD of uORF transcripts? Is it EJC mode, long 3’ UTR mode, both or neither? This will need to be done for each uORF transcript of interest.

Hopefully future research efforts can resolve these and other unknowns surrounding NMD.

## Conclusion

Here I have discussed the NMD pathway in the context of evolution and the many shapes the NMD pathways takes. I have proposed a classification system with four types of NMD pathway, based on the presence/absence of conserved NMD factors. I propose that the classical (type 1) NMD involves UPF1-3, the UPF1-kinase SMG1 and the SMG5/6/7 family. The recent (type 2) and ancient (type 3) loss of SMG1 define the next two types of NMD, while loss of all but UPF1 and perhaps UPF2 define the final type (type 4), where NMD might not actually function at all. It is highly likely that species specific NMD factors have been co-opted in many, if not all, of these types of NMD pathway and are waiting to be discovered. Discussing the evolution and mechanism of NMD within this framework will hopefully aid in the communication of ideas between different model systems used to study NMD and therefore help in knowledge acquisition. Finally, I outline key outstanding questions regarding the mechanism and evolution of the NMD pathway. Focused research efforts to address these issues will certainly help in our overall understanding of the NMD pathway and for us to at last appreciate the true fundamental nature of NMD.

## Data availability

No data are associated with this article.
