# Supplementary material for: The evolution and diversity of the nonsense-mediated mRNA decay pathway
Source: F1000Res. 2018 Nov 22;7:1299. Originally published 2018 Aug 15. [Version 2] doi: 10.12688/f1000research.15872.2 (PMC6173133; doi:10.12688/f1000research.15872.2)
Supplement: Peer review history

# Peer review: Referee response for version 1

**DOI:** 10.5256/f1000research.17330.r37784

**Published:** 2018-09-25

**Authors:** J. Robert Hogg

**Affiliations:** 1 Biochemistry and Biophysics Center, National Heart, Lung, and Blood Institute, National Institutes of Health, Bethesda, MD, USA

The nonsense-mediated mRNA decay pathway is found throughout eukaryotes, where it performs important quality control and regulatory functions. How the pathway originally emerged and has subsequently adapted to diverse eukaryotic transcriptomes is an important but poorly understood question. This manuscript represents a helpful summary of the evidence for compositionally- and mechanistically-distinct NMD pathways in diverse eukaryotes and raises interesting questions that deserve future study.

 Major points:

1. The review raises the question of how RNA decay machinery is recruited to NMD substrate mRNAs in organisms that have lost SMG1. Work from yeast and human cells give two possible answers to this question. First, the Jacobson and Parker labs have presented evidence that budding yeast UPF1 directly interacts with components of the decapping complex, enabling SMG1- and SMG6-independent decay ^1^^,^^2^. Second, the Conti and Muhlemann groups have ^3^^,^^4^ shown that SMG6 can interact with UPF1 in a phosphorylation-independent manner. Their data suggest that UPF1 phosphorylation may be dispensable for SMG6 recruitment but may contribute to activation of its endonucleolytic activity, raising the possibility that a SMG-1 independent pathway could rely on phosphorylation-independent SMG6-UPF1 interactions coupled with a distinct mechanism for activation of SMG6.
2. Figure 3: The schematics in the figure suggest that there is a universal step of mRNP remodeling that involves ribosome displacement from target mRNAs prior to initiation of decay, but it is not clear that this is the case. Previous work from the Baker lab has indicated that budding yeast NMD can initiate on polysome-bound mRNAs, rather than those stripped of ribosomes ^5^. In addition, this figure and Figure 1 should acknowledge that there is evidence that “classical” NMD can also proceed through deadenylation, decapping, and exonucleolytic decay, not just SMG6-mediated cleavage. For other organisms, “cleavage” implies an endonucleolytic step, which in organisms lacking SMG6 is not known to occur. As referenced above, yeast NMD proceeds through decapping, and this should be made clear in the figure.
3. It would be helpful to note that in Drosophila, a much more significant role for SMG1 is uncovered in SMG5 mutants ^6^. This is consistent with the idea that organisms such as Drosophila have developed redundant pathways for NMD. However, this also means that the extent to which “the dependence on SMG1 is not always clear” may be overstated. The fact that SMG1 has been maintained in this organism and can function in at least some contexts should carry greater weight than the failure to observe a strong phenotype in the limited experimental contexts in which it has been examined.
4. Page 6, second paragraph: It is somewhat misleading to state that “transcriptome-wide studies find so few long 3’UTR transcripts that are targeted to NMD.” It is true that there have been differing reports of the extent to which 3’UTR length correlates with decay susceptibility transcriptome-wide, but this is not the same as saying that these studies did not find evidence that substantial numbers of long 3’UTR-containing transcripts are subject to NMD. In addition, it is important to recognize that the Lindeboom *et al.* study cited here examined apparent NMD susceptibility of mRNAs with nonsense mutations, not the scope of long 3’UTR-mediated decay among normal transcripts ^7^.
5. Page 6, second paragraph, continued: The poly-A binding protein-centric model and the UPF1 length-sensing model are not necessarily exclusive. It has been reported that Pab1 and poly-A tails are dispensable for accurate NMD target discrimination in yeast ^8^, but it is possible that UPF1 binding contributes to competition between poly-A binding protein and NMD factors for binding to release factors at the terminating ribosome. Another emerging possibility is that PABP antagonizes UPF1 binding to 3’UTRs, as proposed by Lee *et al.* ^9^.

Minor points:

1. It would be helpful to the reader to reference recent evidence that PNRC2 may function in general decapping but contribute minimally to NMD in vertebrates ^10^.
2. Page 2, second paragraph: Since UPF2 and UPF3 were identified and initially characterized in yeast, in which UPF1 phosphorylation is not known to play a key role in decay, the basis for the statement that “initially, NMD factors were defined by their role in the phosphorylation of UPF1” is unclear.
3. Page 2, first paragraph: The Maquat *et al., *1981 paper was not based on a “mutant screen” but instead observations in human genetic disease ^11^.
4. Figure 1 implies that the roles of UPF2 and UPF3 are dependent on the EJC, but this is not the case — these proteins have been found to be required for decay of NMD substrates lacking 3’UTR introns ^12^^,^^13^.
5. At several points, it is not clear whether the authors use the nomenclature 'SMG5-7' to refer to SMG5, 6, and 7, or just SMG5 and 7, as is the more standard usage. For example, on Page 3, first paragraph, it should be made clear that SMG6 does not function in “recruiting the degradation machinery” but is itself an endonuclease.
6. Page 5, fifth paragraph. It is possible that other PIKK-type kinases other than ATM and ATR are re-purposed to phosphorylate UPF1.
7. Discussion of the Tetrahymena data should acknowledge that at this point the evidence for a role for exon junctions is correlative and remains to be mechanistically investigated. This is an important caveat to the classification system offered by the author ^14^.
8. Page 6, third paragraph: More recent investigations of yeast NMD have not uncovered evidence for a downstream sequence element that contributes significantly to decay target discrimination ^8^^,^^15^. **** ****

I have read this submission. I believe that I have an appropriate level of expertise to confirm that it is of an acceptable scientific standard.

## References

[1] SwisherKDParkerR: Interactions between Upf1 and the decapping factors Edc3 and Pat1 in Saccharomyces cerevisiae. *PLoS One*.2011;6(10) : 10.1371/journal.pone.0026547 e26547 10.1371/journal.pone.0026547 22065998PMC3204985

[2] HeFJacobsonA: Control of mRNA decapping by positive and negative regulatory elements in the Dcp2 C-terminal domain. *RNA*.2015;21(9) : 10.1261/rna.052449.115 1633-47 10.1261/rna.052449.115 26184073PMC4536323

[3] ChakrabartiSBonneauFSchüsslerSEppingerEContiE: Phospho-dependent and phospho-independent interactions of the helicase UPF1 with the NMD factors SMG5-SMG7 and SMG6. *Nucleic Acids Res*.2014;42(14) : 10.1093/nar/gku578 9447-60 10.1093/nar/gku578 25013172PMC4132714

[4] NicholsonPJosiCKurosawaHYamashitaAMühlemannO: A novel phosphorylation-independent interaction between SMG6 and UPF1 is essential for human NMD. *Nucleic Acids Res*.2014;42(14) : 10.1093/nar/gku645 9217-35 10.1093/nar/gku645 25053839PMC4132754

[5] HuWPetzoldCCollerJBakerKE: Nonsense-mediated mRNA decapping occurs on polyribosomes in Saccharomyces cerevisiae. *Nat Struct Mol Biol*.2010;17(2) : 10.1038/nsmb.1734 244-7 10.1038/nsmb.1734 20118937PMC2840703

[6] NelsonJOFörsterDFrizzellKALuschnigSMetzsteinMM: Multiple Nonsense-Mediated mRNA Processes Require Smg5 in Drosophila. *Genetics*.209(4) : 10.1534/genetics.118.301140 1073-1084 10.1534/genetics.118.301140 29903866PMC6063230

[7] LindeboomRGSupekFLehnerB: The rules and impact of nonsense-mediated mRNA decay in human cancers. *Nat Genet*.48(10) : 10.1038/ng.3664 1112-8 10.1038/ng.3664 27618451PMC5045715

[8] MeauxSvan HoofABakerKE: Nonsense-mediated mRNA decay in yeast does not require PAB1 or a poly(A) tail. *Mol Cell*.2008;29(1) : 10.1016/j.molcel.2007.10.031 134-40 10.1016/j.molcel.2007.10.031 18206975PMC2241659

[9] LeeSRPrattGAMartinezFJYeoGWLykke-AndersenJ: Target Discrimination in Nonsense-Mediated mRNA Decay Requires Upf1 ATPase Activity. *Mol Cell*.2015;59(3) : 10.1016/j.molcel.2015.06.036 413-25 10.1016/j.molcel.2015.06.036 26253027PMC4673969

[10] NicholsonPGkratsouAJosiCColomboMMühlemannO: Dissecting the functions of SMG5, SMG7, and PNRC2 in nonsense-mediated mRNA decay of human cells. *RNA*.24(4) : 10.1261/rna.063719.117 557-573 10.1261/rna.063719.117 29348139PMC5855955

[11] MaquatLEKinniburghAJRachmilewitzEARossJ: Unstable beta-globin mRNA in mRNA-deficient beta o thalassemia. *Cell*.1981;27(3 Pt 2) :543-53 610120610.1016/0092-8674(81)90396-2

[12] EberleABStalderLMathysHOrozcoRZMühlemannO: Posttranscriptional gene regulation by spatial rearrangement of the 3' untranslated region. *PLoS Biol*.2008;6(4) : 10.1371/journal.pbio.0060092 e92 10.1371/journal.pbio.0060092 18447580PMC2689704

[13] SinghGRebbapragadaILykke-AndersenJ: A competition between stimulators and antagonists of Upf complex recruitment governs human nonsense-mediated mRNA decay. *PLoS Biol*.2008;6(4) : 10.1371/journal.pbio.0060111 e111 10.1371/journal.pbio.0060111 18447585PMC2689706

[14] TianMYangWZhangJDangHLuXFuCMiaoW: Nonsense-mediated mRNA decay in Tetrahymena is EJC independent and requires a protozoa-specific nuclease. *Nucleic Acids Res*.2017;45(11) : 10.1093/nar/gkx256 6848-6863 10.1093/nar/gkx256 28402567PMC5499736

[15] CelikABakerRHeFJacobsonA: High-resolution profiling of NMD targets in yeast reveals translational fidelity as a basis for substrate selection. *RNA*.23(5) : 10.1261/rna.060541.116 735-748 10.1261/rna.060541.116 28209632PMC5393182

# Response

**Published:** 2018-11-12

**Authors:** James Lloyd

**Affiliations:** University of Western Australia, Australia

Thank you for your thoughtful and insightful reading of my review. You raised a number of important points that I have tried to address in version 2 of my review article. Some of the points I directly address are:

Second, the Conti and Muhlemann groups have ^3,4^ shown that SMG6 can interact with UPF1 in a phosphorylation-independent manner.

It would be helpful to note that in Drosophila, a much more significant role for SMG1 is uncovered in SMG5 mutants ^6^.

*This is a very important point and I think that this points to a possible mechanism to explain how some species can have an active NMD pathway without SMG1. When we combine this with recent yeast work finding direct interactions between EBS1 and NMD4, and UPF1, this suggests that in many cases, phosphorylation is not needed for NMD. This supports a model that in mammals phosphorylation of UPF1 increases if NMD is stalled, suggesting that it acts to increase the recruitment of degradation factors (https://www.nature.com/articles/ncomms12434), potentially in NMD limiting conditions or for tricky to degrade transcripts.*

The poly-A binding protein-centric model and the UPF1 length-sensing model are not necessarily exclusive. It has been reported that Pab1 and poly-A tails are dispensable for accurate NMD target discrimination in yeast ^8^, but it is possible that UPF1 binding contributes to competition between poly-A binding protein and NMD factors for binding to release factors at the terminating ribosome. Another emerging possibility is that PABP antagonizes UPF1 binding to 3’UTRs, as proposed by Lee et al. ^9^.

*This is a fair point and I did not mean to suggest that the two models were mutually exclusive in the first version. Also I think that Lee et al. is a great reference for me to include, thank you for bringing it to my attention!*

It would be helpful to the reader to reference recent evidence that PNRC2 may function in general decapping but contribute minimally to NMD in vertebrates ^10^.

*Thank you for raising this point about PNRC2, as did other referees and I have removed mention of it from my review for the sake of simplicity.*

Figure 1 implies that the roles of UPF2 and UPF3 are dependent on the EJC, but this is not the case — these proteins have been found to be required for decay of NMD substrates lacking 3’UTR introns ^12,13^.

*Yes this is a very important point and I did not mean to give that impression with this figure. I have removed the EJC from Figure 3 and I have mentioned in the legend of Figure 1 that NMD can happen without an EJC present in the now submitted version 2 of this manuscript.*

It is possible that other PIKK-type kinases other than ATM and ATR are re-purposed to phosphorylate UPF1.

*The only other kinase active PIKK in Arabidopsis is TOR, which I have now included in the submitted version 2 of this review.*

More recent investigations of yeast NMD have not uncovered evidence for a downstream sequence element that contributes significantly to decay target discrimination ^8,15^.

*I have now removed the section of DSE from the submitted version 2 of this review.*

# Peer review: Referee response for version 1

**DOI:** 10.5256/f1000research.17330.r38016

**Published:** 2018-09-24

**Authors:** Niels H. Gehring

**Affiliations:** 1 Institute for Genetics, Department of Biology, University of Cologne, Cologne, North Rhine-Westphalia, Germany

In this review article James P. B. Lloyd summarizes the evolution of NMD in various eukaryotic organisms. He describes the process of NMD in general as well as the detection of NMD substrates by the NMD machinery and discusses the functions of several NMD factors. As a novelty, he introduces a new classification of NMD pathways based on the presence of NMD factors in different organisms.

 In my view, this is a well-written review that takes a refreshing new look at evolutionary aspects of NMD. However, one should keep in mind that the absence of a certain NMD pathway does not necessarily mean that it really does not exist in a particular organism. A good example for this is NMD in Drosophila, which was thought to be EJC-independent based on initial publications. However, there is now evidence that certain NMD substrates in Drosophila are degraded in an EJC-dependent manner. It is therefore possible that the classification of NMD pathways will change with future publications.

 Additional comments:

 Page 4, last paragraph on the right side: The author probably does not mean ETS1, but EST1 (Reichenbach *et al.*, 2003 ^1^). Furthermore, a second SMG6 homologue in yeast may be NMD4, which has been originally identified in a yeast-2-hybrid screen (He and Jacobson, 1995 ^2^) and was recently described by Dehecq *et al.* (Dehecq *et al.*, 2018 ^3^) to be associated with UPF1.

 Page 4, last paragraph on the right side and page 5, first paragraph on the left side: Wang *et al.* (Wang *et al.*, 2006 ^4^) and de Pinto *et al.* (de Pinto *et al.*, 2004 ^5^) have reported that UPF1 and UPF2 are phosphoproteins in yeast. However, it is not clear which kinase is responsible for the phosphorylation of UPF1 and UPF2 and whether the phosphorylation plays a functional role during NMD in yeast.

 Page 5, penultimate paragraph on the right side: It is strongly debated whether PNRC2 is an NMD factor (Nicholson *et al.*, 2018 ^6^). Therefore, PNRC2 is not a good example for a vertebrate-specific NMD factor.

 Page 6, last paragraph on the right side: the meaning of the sentence “These adaptations ensure that retention of these efficiently-spliced introns would not be repeatedly translation” is not clear to me.

 Page 7, question 2: It is known that SMG6 can interact with UPF1 also in a phosphorylation-independent manner (Nicholson *et al.*, 2014 ^7^ and Chakrabarti *et al.*, 2014 ^8^). This could explain how NMD substrates are degraded in the absence of SMG1 or when S/TQ dipeptides are depleted.

I have read this submission. I believe that I have an appropriate level of expertise to confirm that it is of an acceptable scientific standard.

## References

[1] ReichenbachPHössMAzzalinCNabholzMBucherPLingnerJ: A Human Homolog of Yeast Est1 Associates with Telomerase and Uncaps Chromosome Ends When Overexpressed. *Current Biology*.2003;13(7) : 10.1016/S0960-9822(03)00173-8 568-574 10.1016/S0960-9822(03)00173-8 12676087

[2] HeFJacobsonA: Identification of a novel component of the nonsense-mediated mRNA decay pathway by use of an interacting protein screen. *Genes & Development*.1995;9(4) : 10.1101/gad.9.4.437 437-454 10.1101/gad.9.4.437 7883168

[3] DehecqMDecourtyLNamaneAProuxCKanaanJLe HirHJacquierASaveanuC: Detection and Degradation of Nonsense-mediated mRNA Decay Substrates Involve Two Distinct Upf1-bound Complexes. *bioRxiv*.2018; 10.1101/266833 10.1101/266833 PMC621328530275269

[4] WangWCajigasIJPeltzSWWilkinsonMFGonzálezCI: Role for Upf2p phosphorylation in Saccharomyces cerevisiae nonsense-mediated mRNA decay. *Mol Cell Biol*.2006;26(9) : 10.1128/MCB.26.9.3390-3400.2006 3390-400 10.1128/MCB.26.9.3390-3400.2006 16611983PMC1447418

[5] de PintoBLippolisRCastaldoRAltamuraN: Overexpression of Upf1p compensates for mitochondrial splicing deficiency independently of its role in mRNA surveillance. *Molecular Microbiology*.2004;51(4) : 10.1046/j.1365-2958.2003.03889.x 1129-1142 10.1046/j.1365-2958.2003.03889.x 14763985

[6] NicholsonPGkratsouAJosiCColomboMMühlemannO: Dissecting the functions of SMG5, SMG7, and PNRC2 in nonsense-mediated mRNA decay of human cells. *RNA*.24(4) : 10.1261/rna.063719.117 557-573 10.1261/rna.063719.117 29348139PMC5855955

[7] NicholsonPJosiCKurosawaHYamashitaAMühlemannO: A novel phosphorylation-independent interaction between SMG6 and UPF1 is essential for human NMD. *Nucleic Acids Res*.2014;42(14) : 10.1093/nar/gku645 9217-35 10.1093/nar/gku645 25053839PMC4132754

[8] ChakrabartiSBonneauFSchüsslerSEppingerEContiE: Phospho-dependent and phospho-independent interactions of the helicase UPF1 with the NMD factors SMG5-SMG7 and SMG6. *Nucleic Acids Res*.2014;42(14) : 10.1093/nar/gku578 9447-60 10.1093/nar/gku578 25013172PMC4132714

# Response

**Published:** 2018-11-12

**Authors:** James Lloyd

**Affiliations:** University of Western Australia, Australia

I am truly grateful for your careful reading and thoughtful feedback of my review. I have now submitted a version 2 of this article that updated the text and figures with many of the insights you and the other referees offered. Below are some highlights of things that I address in the updated manuscript:

one should keep in mind that the absence of a certain NMD pathway does not necessarily mean that it really does not exist in a particular organism. A good example for this is NMD in Drosophila, which was thought to be EJC-independent based on initial publications. However, there is now evidence that certain NMD substrates in Drosophila are degraded in an EJC-dependent manner. It is therefore possible that the classification of NMD pathways will change with future publications.

*I completely agree with this point and I hope that the readers will take this message away with them after reading my review.*

The author probably does not mean ETS1, but EST1

*Yes, thank you for catching this.*

Furthermore, a second SMG6 homologue in yeast may be NMD4, which has been originally identified in a yeast-2-hybrid screen (He and Jacobson, 1995 ^2^) and was recently described by Dehecq et al. (Dehecq et al., 2018 ^3^) to be associated with UPF1.

*Thank you for bringing Dehecq et al. to my attention! I think that this article represents very important work and I have now included much discussion of it and its implications in the submitted version 2 of my article.*

 penultimate paragraph on the right side: It is strongly debated whether PNRC2 is an NMD factor

*Thank you for raising this point about PNRC2, as did other referees and I have removed mention of it from my review for the sake of simplicity.*

It is known that SMG6 can interact with UPF1 also in a phosphorylation-independent manner (Nicholson et al., 2014 ^7^ and Chakrabarti et al., 2014 ^8^). This could explain how NMD substrates are degraded in the absence of SMG1 or when S/TQ dipeptides are depleted.

*Yes, I think that this is a really great point and I discuss this at length in the submitted version 2 of the review. Thank you for raising this point.*

# Peer review: Referee response for version 1

**DOI:** 10.5256/f1000research.17330.r37786

**Published:** 2018-09-21

**Authors:** Damien Garcia

**Affiliations:** 1 Institute of Molecular Biology of Plants(IBMP), University of Strasbourg, Strasbourg, France

In this review, the author suggests a novel NMD classification system based on the evolutionary conservation of known NMD factors among different species. The author also discusses a possible driving force for the apparition of NMD. This is a well-written and structured manuscript considering NMD under an interesting evolutionary point of view. It is of broad interest for the NMD community, and it mentions several fundamental questions currently unanswered in the NMD field. Nevertheless, a few modifications of the figures and the addition of some very important references and concepts could make it easier to read and broaden the general interest of the review, as detailed in the following paragraphs.

**Major comments:**

  

1. In Figure 1, ****the model of mammalian NMD indicates cleavage of the NMD target downstream of the PTC. It doesn’t indicate the distinct roles of SMG6 and SMG5/7; it would be important here to indicate the distinct involvement of SMG6 in the cleavage activity and the role of SMG5/7 in decay factors recruitment. These two routes for decay should clearly appear in Figure 1.
2. In Figure 2, the definition of Type 1/2/3/4 NMD should already be mentioned in the figure legends (e.g. Type 1: classical SMG1 dependent NMD, Type 2: recent loss of SMG1 with conserved phosphorylation, Type 3: ancient loss of SMG1 with loss of UPF1 S/Q phosphorylation, Type 4: Heavily derived NMD).
3. In the paragraph 'Defining NMD targets' **, **the author cites the 2016 paper on the NMD protection effect of PTB1; an earlier study describing similar protection effect of Pub1 in yeast should also be cited (Ruiz-Echevarria and Peltz, 2000 ^1^).
4. In the same paragraph, the author focuses on Baker’s yeast DSE, stimulating NMD. The author should also mention a recent paper in yeast describing that poor translation efficiency is a major criteria for NMD targeting (Celik et al., 2017 ^2^). This major result could potentially explain not yet understood deregulations observed in several other species upon NMD knockdown.
5. It was proposed that invading RNAs of external origin, including TEs and viruses, could be a driving force for NMD apparition and evolution - this should be mentioned in the paragraph on the possible origin of NMD (Hamid and Makeyev, 2016 ^3^).

 

**Minor comments:**

  

1. UPF1 might have other essential functions beyond NMD, as observed for UPF3b in mammals, involved in translation termination (Neu-Yilik et al., 2017 ^4^), which could explain its presence in some species without any other known NMD factors. This could be mentioned in the corresponding section.
2. As branches of NMD exist without the need of UFP2/UPF3, it suggests that NMD could be active with only UPF1. This possibility should be discussed/mentioned when describing Type 4 species depleted of UPF2 and UPF3.
3. In addition to ATR/ATM, the author could mention TOR or TRRAP kinases as described in (Lloyd and Davies, 2013 ^5^), as possible kinase replacements for SMG1.
4. In Figure 3, the author should add precisions on endonucleolytic cleavage and decay factor recruitment for **(A)**, and decay factor recruitment only for the others. The different types of NMD defined in Figure 2 should be mentioned again here in Figure 3, namely: Type 1, Type 2, Type 3 and Type 4.

I have read this submission. I believe that I have an appropriate level of expertise to confirm that it is of an acceptable scientific standard.

## References

[1] Ruiz-EchevarríaMJPeltzSW: The RNA binding protein Pub1 modulates the stability of transcripts containing upstream open reading frames. *Cell*.2000;101(7) :741-51 1089274510.1016/s0092-8674(00)80886-7

[2] CelikABakerRHeFJacobsonA: High-resolution profiling of NMD targets in yeast reveals translational fidelity as a basis for substrate selection. *RNA*.23(5) : 10.1261/rna.060541.116 735-748 10.1261/rna.060541.116 28209632PMC5393182

[3] HamidFMMakeyevEV: Exaptive origins of regulated mRNA decay in eukaryotes. *Bioessays*.38(9) : 10.1002/bies.201600100 830-8 10.1002/bies.201600100 27438915PMC5031192

[4] Neu‐YilikGRaimondeauEEliseevBYeramalaLAmthorBDeniaudAHuardKKerschgensKHentzeMSchaffitzelCKulozikA: Dual function of UPF3B in early and late translation termination. *The EMBO Journal*.2017;36(20) : 10.15252/embj.201797079 2968-2986 10.15252/embj.201797079 PMC564191328899899

[5] LloydJPDaviesB: SMG1 is an ancient nonsense-mediated mRNA decay effector. *Plant J*.2013;76(5) : 10.1111/tpj.12329 800-10 10.1111/tpj.12329 24103012

# Response

**Published:** 2018-11-12

**Authors:** James Lloyd

**Affiliations:** University of Western Australia, Australia

I am thankful for you carefully reading my review and giving great suggestions for its improvement. I have now submitted a version 2 of this article that updated the text and figures with many of the insights you and the other referees offered. Below are some highlights of things that I address in the updated manuscript:

involvement of SMG6 in the cleavage activity and the role of SMG5/7 in decay factors recruitment. These two routes for decay should clearly appear in Figure 1.

*This is a great point and I have tried to address this in the submitted version 2 of the review.* 

In Figure 2, the definition of Type 1/2/3/4 NMD should already be mentioned in the figure legends

*I have now added this in the submitted version 2 of this review.*

In the paragraph 'Defining NMD targets', the author cites the 2016 paper on the NMD protection effect of PTB1; an earlier study describing similar protection effect of Pub1 in yeast should also be cited

*Thank you for bringing this to my attention and it is now included in the submitted version 2 of this review.*

It was proposed that invading RNAs of external origin, including TEs and viruses, could be a driving force for NMD apparition and evolution - this should be mentioned in the paragraph on the possible origin of NMD (Hamid and Makeyev, 2016 ^3^).

*I have now cited this work in the submitted version 2 of this review.*

UPF1 might have other essential functions beyond NMD, as observed for UPF3b in mammals, involved in translation termination (Neu-Yilik et al., 2017 ^4^), which could explain its presence in some species without any other known NMD factors. This could be mentioned in the corresponding section.

*Great point and I have now mentioned this in the submitted version 2 of this review.*

As branches of NMD exist without the need of UFP2/UPF3, it suggests that NMD could be active with only UPF1. This possibility should be discussed/mentioned when describing Type 4 species depleted of UPF2 and UPF3.

*Agreed and this is now mentioned in the submitted version 2 of this review.*

In addition to ATR/ATM, the author could mention TOR or TRRAP kinases as described in (Lloyd and Davies, 2013 ^5^), as possible kinase replacements for SMG1.

*Good point, I have now added TOR to the submitted version 2 of this review. I did not add TRRAP because TRRAP has been reported to be a kinase dead member of the family (https://www.sciencedirect.com/science/article/pii/S0092867400814798?via%3Dihub).*

# Peer review: Referee response for version 1

**DOI:** 10.5256/f1000research.17330.r37408

**Published:** 2018-08-30

**Authors:** Wei Miao

**Affiliations:** 1 Key Laboratory of Aquatic Biodiversity and Conservation, Institute of Hydrobiology (IHB), Chinese Academy of Sciences (CAS), Wuhan, China

In this manuscript, the author discussed the diversity of the NMD pathway according to the variation of NMD factors in different eukaryotes, and briefly summarized some popular NMD models. Furthermore, the author also discussed the relationship between intron gain/loss and NMD. Several interesting and unsolved questions in this field were also mentioned by the end of the manuscript. Interestingly, the author came up with a novel classification system (although it needs to be further modified, see below) to classify NMD mechanisms into different types based on the new criterion. Namely, it classifies NMD mechanisms according to the presence or absence of key factors that related to the well-characterized Upf1 C-terminus phosphorylation events. The manuscript is clearly written, and this work is likely to be of general interest to the NMD field. My main criticism is about the novel classification system.

 Major

1. As it is still unclear whether excavates have functional NMD, the type 4 NMD may not need to be taken into account. Or question marks should be included in the figure 2 and figure 3D, and in the main text. 
2. Type 1 NMD seems to be SMG1-dependent and EJC-dependent (see figure 3A). Hence it cannot be exemplified by C. elegans (EJC-independent NMD). The author could modify the figure a little. For example, one could draw EJC with a dashed line to indicate it is dispensable for NMD in some organisms, like C. elegans. Besides, the presence of Smg1 and Upf1 orthologs in N. gruberi does not necessarily mean it possesses a functional NMD pathway, thus I suggest not consider it has type 1 NMD. Or, as I mentioned above, excavates need not be discussed too much.
3. As mentioned by the author, some organisms (e.g., D. melanogaster and D. rerio) are considered to have SMG1-dependent type 1 NMD, yet their SMG1 proteins are dispensable for NMD activation and therefore similar to the type 2 NMD. I am wondering whether this issue can be solved by defining another type of NMD, namely in between type 1 and type 2. Besides, an additional figure (e.g., figure 3 in (Lareau and Brenner, 2015 ^1^) could be provided to show the evolutionary relationship between different types of NMD. 
4. Figure 2 needs to be modified. Clearly, solely based on the pattern of these icons (NMD proteins), readers may get confused why organisms with the same pattern are not classified into the same group. For example, like C. rubella, Tetrahymena also has a red square and a blue triangle. However, they are classified into different types. Although, in this case, it can be easily solved by adding another icon to indicate Upf1 with phosphorylatable S/TQ motifs. Additional modifications are required to let readers understand, for example, why Dikarya and Mirosporia are considered as type 1, but not type 2. 

Minor

1. Page 2, left panel, lane 10. Ciliates should be mentioned here as well, because NMD was also proved to be required for regulating many of their transcripts (Jaillon et al. 2008 ^2^, Tian et al. 2017 ^3^).
2. Page 2, right panel, lanes 19 – 20. I suggest deleting this sentence. Or, I would rather say that, even in animals, NMD factors were simply defined by their requirement for NMD. Because phosphorylation of Upf1 seems to be not essential for eliciting NMD in some animals (e.g., SMG1-independent NMD in fruit flies and zebrafish). 
3. Page 2, figure 1, the bottom panel. I suggest moving Upf2, Upf1 and its associated proteins (especially the endonuclease Smg6) to the left side of the EJC, close to the endonucleolytic cleavage site.
4. Page 2, figure 1, the title of figure legend. The word “Animals” is probably too general, hence the author may want to replace it with another word (e.g., vertebrates). Because it is known that EJC is not required or dispensable for NMD in some invertebrates (Longman et al. 2007 ^4^, Gatfield et al. 2003 ^5^). 
5. Page 2, figure 1, the last sentence of the figure legend. Replace “phoso-UPF1” with “phosphor-UPF1” or “phos-UPF1”.
6. Page 3, right panel, lane 8. Smg7 recruits CCR4–NOT deadenylase complex instead of exonuclease (Loh et al. 2013 ^6^).
7. Page 3, left panel, lane 14. Reference 34 (Rosains and Mango, 2012 ^7^) suggests that Smg8 is not required for NMD in C. elegans.
8. Page 3, left panel, lane 28. Change the word “animals”, see comments #3.
9. Page 3, left panel, the first paragraph of the section “Variations on a common pathway”. Since it is still unclear whether NMD exists in excavates, it is inappropriate to say that they have “the most divergent NMD pathway”. Some following sentences in this paragraph are also against the existence of NMD pathway in excavates. To support the hypothesis “a complex NMD pathway involving… in the last eukaryotic common ancestor”, the author could start the discussion from plant NMD mechanism, and then use the existence of orthologs of NMD core factors in excavates as a supporting evidence.
10. Page 4, figure 3 legend. “In T, it has been shown” should be “In T. brucei …”.
11. Page 5, left panel, lanes 2 – 3 and lanes 20 – 21. The author should mention that the interaction between Upf1 and Smg6 can also be phosphorylation-independent (Chakrabarti et al. 2014 ^8^).
12. Page 5 and many other places. It is better to use a unified way to indicate different organisms (groups). For example: change “C. elegans, humans, and moss” to “C. elegans, H. sapiens and P. patens” or “worms, humans and moss”. 
13. Page 5, right panel, lane 5. Is there experimental evidence to support that S/TQ sites have undergone phosphorylation? If not, I suggest replacing “S/TQ dipeptide phosphorylation sites” with “phosphorylatable S/TQ motifs”. 
14. Page 5, right panel, “PNRC2 is a vertebrate-specific NMD factor”. The author may want to remove this sentence because a recent study has shown that PNRC2 may not be required for NMD (Nicholson et al. 2018 ^9^).
15. Page 6, left panel, lanes 19 – 21. The author should modify this sentence a little. Despite the splice junctions downstream of the stop codon (DSJ) are enriched in potential Tetrahymena NMD targets (Tian et al. 2017 ^3^), it is still insufficient to conclude that “NMD in T. thermophila is dependent on DSJ”. 
16. Page 6, left panel, bottom. The description of DSE model could be removed because evidence from a few studies are against this model (for example (Meaux et al. 2008 ^10^).
17. Page 6, right panel, lane 12. The fungus N. crassa should also be mentioned here, due to the requirement of EJC for its NMD pathway. 
18. Page 6, right panel, section “The origins of NMD”, the second sentence. The word “clear” is too strong here. 
19. Page 6, right panel, section “The origins of NMD”. Firstly, the author may want to change the subtitle of this section. In this section, a clear answer to origins of NMD is not given, and discussions are mainly about the relationship between NMD and the intron evolution. Secondly, when discussing the relationship between intron evolution and (the evolution of) NMD, an earlier study from Michael Lynch and Avinash Kewalramani is deserved to be mentioned here (Lynch et al. 2003 ^11^).
20. Page 7, left panel, section “unanswered questions”. Regarding the question 2, the author should consider the existence of phosphorylation-independent interaction between Upf1 and Smg6. The question 4 should be modified as the existence of “an EJC mode of PTC recognition” in Tetrahymena has not been proven yet.

I have read this submission. I believe that I have an appropriate level of expertise to confirm that it is of an acceptable scientific standard.

## References

[1] LareauLFBrennerSE: Regulation of splicing factors by alternative splicing and NMD is conserved between kingdoms yet evolutionarily flexible. *Mol Biol Evol*.2015;32(4) : 10.1093/molbev/msv002 1072-9 10.1093/molbev/msv002 25576366PMC4379411

[2] JaillonOBouhoucheKGoutJFAuryJMNoelBSaudemontBNowackiMSerranoVPorcelBMSégurensBLe MouëlALepèreGSchächterVBétermierMCohenJWinckerPSperlingLDuretLMeyerE: Translational control of intron splicing in eukaryotes. *Nature*.2008;451(7176) : 10.1038/nature06495 359-62 10.1038/nature06495 18202663

[3] TianMYangWZhangJDangHLuXFuCMiaoW: Nonsense-mediated mRNA decay in Tetrahymena is EJC independent and requires a protozoa-specific nuclease. *Nucleic Acids Res*.2017;45(11) : 10.1093/nar/gkx256 6848-6863 10.1093/nar/gkx256 28402567PMC5499736

[4] LongmanDPlasterkRHJohnstoneILCáceresJF: Mechanistic insights and identification of two novel factors in the C. elegans NMD pathway. *Genes Dev*.2007;21(9) : 10.1101/gad.417707 1075-85 10.1101/gad.417707 17437990PMC1855233

[5] GatfieldDUnterholznerLCiccarelliFDBorkPIzaurraldeE: Nonsense-mediated mRNA decay in Drosophila: at the intersection of the yeast and mammalian pathways. *EMBO J*.2003;22(15) : 10.1093/emboj/cdg371 3960-70 10.1093/emboj/cdg371 12881430PMC169044

[6] LohBJonasSIzaurraldeE: The SMG5-SMG7 heterodimer directly recruits the CCR4-NOT deadenylase complex to mRNAs containing nonsense codons via interaction with POP2. *Genes Dev*.2013;27(19) : 10.1101/gad.226951.113 2125-38 10.1101/gad.226951.113 24115769PMC3850096

[7] RosainsJMangoSE: Genetic characterization of smg-8 mutants reveals no role in C. elegans nonsense mediated decay. *PLoS One*.2012;7(11) : 10.1371/journal.pone.0049490 e49490 10.1371/journal.pone.0049490 23166684PMC3500306

[8] ChakrabartiSBonneauFSchüsslerSEppingerEContiE: Phospho-dependent and phospho-independent interactions of the helicase UPF1 with the NMD factors SMG5-SMG7 and SMG6. *Nucleic Acids Res*.2014;42(14) : 10.1093/nar/gku578 9447-60 10.1093/nar/gku578 25013172PMC4132714

[9] NicholsonPGkratsouAJosiCColomboMMühlemannO: Dissecting the functions of SMG5, SMG7, and PNRC2 in nonsense-mediated mRNA decay of human cells. *RNA*.24(4) : 10.1261/rna.063719.117 557-573 10.1261/rna.063719.117 29348139PMC5855955

[10] MeauxSvan HoofABakerKE: Nonsense-mediated mRNA decay in yeast does not require PAB1 or a poly(A) tail. *Mol Cell*.2008;29(1) : 10.1016/j.molcel.2007.10.031 134-40 10.1016/j.molcel.2007.10.031 18206975PMC2241659

[11] LynchMKewalramaniA: Messenger RNA surveillance and the evolutionary proliferation of introns. *Mol Biol Evol*.2003;20(4) : 10.1093/molbev/msg068 563-71 10.1093/molbev/msg068 12654936

# Response

**Published:** 2018-11-12

**Authors:** James Lloyd

**Affiliations:** University of Western Australia, Australia

Thank you for reading my review and giving so many insights into how I can improve my manuscript. I have now submitted a version 2 of this article that updated the text and figures with many of the insights you and the other referees offered. Below are some highlights of things that I address in the updated manuscript:

As it is still unclear whether excavates have functional NMD, the type 4 NMD may not need to be taken into account

*Certainly and I have tried to make this clear in the submitted version 2 of this review.*

Type 1 NMD seems to be SMG1-dependent and EJC-dependent (see figure 3A). Hence it cannot be exemplified by C. elegans (EJC-independent NMD).

*I did not mean to give that impression so I have removed the EJC from Figure 3 of the submitted version 2 of this review. Also, while no reports of EJC involvement in C. elegans are published, I do not think we have enough data to state whether NMD in C. elegans is really EJC-independent and I look forward to future work that might add to this.*

Figure 2 needs to be modified. Clearly, solely based on the pattern of these icons (NMD proteins), readers may get confused why organisms with the same pattern are not classified into the same group. For example, like C. rubella, Tetrahymena also has a red square and a blue triangle. However, they are classified into different types. Although, in this case, it can be easily solved by adding another icon to indicate Upf1 with phosphorylatable S/TQ motifs. Additional modifications are required to let readers understand, for example, why Dikarya and Mirosporia are considered as type 1, but not type 2. 

*Thank you for raising some issues with Figure 2. I have now corrected some of my mistakes of misclassification in this figure. I was not satisfied with any of my attempts to differentiate between UPF1 proteins of Type 2 and 3 so that I have left unchanged but I was a good point that you raise.*

“PNRC2 is a vertebrate-specific NMD factor”. The author may want to remove this sentence because a recent study has shown that PNRC2 may not be required for NMD

*Thank you for raising this point about PNRC2, as did other referees and I have removed mention of it from my review for the sake of simplicity.*

The description of DSE model could be removed because evidence from a few studies are against this model

*This is a great point and I have now removed the section of DSE from the submitted version 2 of this review.*
